# The Effect of Dosage, Gestational Age and Splenectomy on Anti-IgM Interception of Prenatal B-cell Development in Sheep

**DOI:** 10.1080/10446670310001598500

**Published:** 2003-03

**Authors:** P. McCullagh, C. McL. Press, S. J. McClure, H. J. Larsen, T. Landsverk

**Affiliations:** 1 John Curtin School of Medical Research Australian National University Canberra ACT 2600 Australia; 2 Department of Morphology Genetics and Aquatic Biology Norwegian School of Veterinary Science P.O. Box 8146, Dep. 0033 Oslo Norway; 3 CSIRO Livestock Industries McMaster Laboratory Armidale 2350 NSW Australia; 4 Department of Pharmacology Microbiology and Food Hygiene Norwegian School of Veterinary Science Oslo Norway

## Abstract

The administration of a single bolus of anti-IgM antibody to foetal lambs early in pregnancy produces prolonged B-cell depletion. The present study investigated this depletion by examining the effect, on B-cell development in the ileal Peyer's patches, of varying the timing and dosage of antibody administration and by supplementing anti-IgM with surgical splenectomy. The capacity of a 1 mg bolus of anti-IgM to deplete Peyer's patches of B cells was lost if its administration was deferred until two thirds of the way through pregnancy, but persisted beyond this time if weight-adjusted doses were used. Splenectomy of the foetus performed at an earlier age failed to extend the age at which a 1 mg dose of antibody remained effective. As the concentration of murine immunoglobulin in foetal serum was greatly reduced after 21 days, it is inferred that ongoing suppression of B-cell development is not dependent on the continued presence of murine immunoglobulin. The enduring nature of suppression could be attributable to a limited period during which differentiation of B cells from stem cells normally occurs, although further studies will be needed to investigate this and other possible explanations for the effect of anti-IgM treatment on prenatal B-cell development in sheep.


*Clinical & Developmental Immunology*, March 2003, VOL. 10 (1), pp. 19–26

Taylor & Francis healthsciences

### The Effect of Dosage, Gestational Age and Splenectomy on Anti-IgM Interception of Prenatal B-cell Development in Sheep

P. McCULLAGH[a], C.McL. PRESS[b,*], S.J. McCLURE[c], H.J. LARSEN[d] and T. LANDSVERK[b]

[a]*John Curtin School of Medical Research, Australian National University, Canberra, ACT 2600, Australia;* [b]*Department of Morphology, Genetics and Aquatic Biology, Norwegian School of Veterinary Science, P.O. Box 8146, Dep. 0033, Oslo, Norway;* [c]*CSIRO Livestock Industries McMaster Laboratory, Armidale, NSW 2350, Australia;* [d]*Department of Pharmacology, Microbiology and Food Hygiene, Norwegian School of Veterinary Science, Oslo, Norway*

The administration of a single bolus of anti-IgM antibody to foetal lambs early in pregnancy produces prolonged B-cell depletion. The present study investigated this depletion by examining the effect, on B-cell development in the ileal Peyer's patches, of varying the timing and dosage of antibody administration and by supplementing anti-IgM with surgical splenectomy. The capacity of a 1 mg bolus of anti-IgM to deplete Peyer's patches of B cells was lost if its administration was deferred until two thirds of the way through pregnancy, but persisted beyond this time if weight-adjusted doses were used. Splenectomy of the foetus performed at an earlier age failed to extend the age at which a 1 mg dose of antibody remained effective. As the concentration of murine immunoglobulin in foetal serum was greatly reduced after 21 days, it is inferred that ongoing suppression of B-cell development is not dependent on the continued presence of murine immunoglobulin. The enduring nature of suppression could be attributable to a limited period during which differentiation of B cells from stem cells normally occurs, although further studies will be needed to investigate this and other possible explanations for the effect of anti-IgM treatment on prenatal B-cell development in sheep.

*Keywords*: B-cell development; Anti-IgM; Peyer's patch; Foetus; Sheep

### INTRODUCTION

The demonstration that a single injection of an anti-IgM monoclonal early in gestation depleted the B-cell populations in foetal sheep raised a number of questions as to the nature of prenatal B-cell development in sheep (Press *et al.*, 1996; 1998). In laboratory mammals such as mice and rabbits, maintenance of the suppression of B-cell development in the neonate requires repeated injections of anti-IgM antibodies (Gordon, 1979; Cooper *et al.*, 1980). It has been shown that the modulation of the IgM receptor of neonatal B cells by antibodies leads to aborted B-cell development (Cooper *et al.*, 1980) and studies in 8-week-old lambs have also shown that ileal Peyer's patch B cells can be induced to undergo apoptosis or switch off proliferation by surface immunoglobulin ligation (Griebel *et al.*, 1991; Motyka *et al.*, 1995). The conclusion from an initial series of experiments with B-cell depleted lambs was that the administration of anti-IgM to foetal sheep at 63 days of gestation (dg; gestation in sheep is 150 days) had inhibited cell division and/or induced cell death in surface IgM+ cells (Press *et al.*, 1996).

The requirement for repeated injections of anti-IgM antibody to maintain B-cell depletion in mice arises because on-going antigen independent differentiation of stem cells to B cells occurs first in the foetal liver and then in the bone marrow of the adult (Alt *et al.*, 1987). The rate of production of pre-B cells does not differ between anti-IgM-treated mice and controls given normal rabbit serum indicating that these precursors are not susceptible to this antibody (Opstelten and Osmond, 1985). In foetal sheep, in contrast to the effects observed in mice, a single injection of anti-IgM antibody at 63 dg maintained a marked depletion of surface IgM+ cells for 80 days. One possible explanation for this long-standing effect of anti-IgM treatment was that an IgM+ cell population crucial for ongoing B-cell production was inactivated or eliminated. The ileal Peyer's patch is responsible for the production of the vast majority of B cells in sheep and active lymphopoiesis begins in this site at around 100 dg (Reynolds and Morris, 1983; Press *et al.*, 1992). The earliest IgM+ B cells have been detected in the foetal spleen at 45–50 dg (Maddox *et al.*, 1987a; Press *et al.*, 1993). At 63 dg, B cells are prominent in the spleen and it was suggested that the removal of this population was responsible for the marked depletion of B cells seen in foetuses at around 140 dg (Press *et al.*, 1996). However, a subsequent experiment in which surgical splenectomy

*Corresponding author. Tel.: +47-22597037. Fax: +47-22964764. E-mail: charles.press@veths.no

ISSN 1740-2522 print/ISSN 1740-2530 online © 2003 Taylor & Francis Ltd
DOI: 10.1080/10446670310001598500

was undertaken at 56 dg disposed of this possibility with the finding that the morphology of the lymphoid tissue of operated foetuses was substantially normal when examined near term (Press *et al.*, 2001). This clear-cut demonstration of the non-equivalence of anti-IgM administration and splenectomy was interpreted as indicating that other, unidentified parts of the lymphoid system could compensate for the loss of the spleen. In contrast, it would appear that antibody administration either inactivated an essential cell population and/or exerted a continuing effect that was sufficient to inactivate potential replacement cells.

Reynaud *et al.* (1991) suggested that the limited pattern of light chain rearrangement observed in the ileal Peyer's patch of sheep indicated that immunoglobulin gene rearrangement only occurred during a narrow window of development. The experiments with B-cell depleted sheep have drawn attention to the nature of this window in B-cell development. An important aspect of this experimental model that was not addressed in the initial series of experiments (Press *et al.*, 1996; 1998), was the duration of the effect of anti-IgM administration. The catabolic rate of ovine gamma globulin experimentally introduced in foetal ruminants is extremely low (Fukumoto and Brandon, 1985). This has been attributed to the agammaglobulinaemic status of these foetal animals, on the basis of the linkage that exists between catabolic rate at any time and the serum globulin level (Fukumoto and Brandon, 1985). However, it has also been demonstrated that sufficient lysosomal enzyme activity exists in the foetal liver, spleen, lymph nodes and plasma to account for a significant amount of protein degradation (Fukumoto and Brandon, 1985). Thus, the kinetics of the single bolus of injected murine antibody in foetal lambs remain uncertain.

This paper reports experiments in which examination of the consequences of variations in dosage and gestational age at administration of anti-IgM, and of combining splenectomy with an antibody administration regime was undertaken. These experiments were performed to assist in identifying the detailed mechanism of antibody action. The interpretation of these observations was supplemented by observation of the duration of persistence of administered murine gamma globulin in the foetal lamb circulation.

### MATERIALS AND METHODS

### Animals and Tissue Collection

Foetal lambs of known gestational age were obtained from timed matings of Merino ewes. To achieve this, intravaginal sponges containing flugestone acetate (40 mg Chronogest, Laboratiore Pharmacetique, Porges, France) were placed in the ewes to be mated. Eleven days later, sponges were removed and ewes penned with rams wearing a harness with an attached crayon that marked each ewe at the time of joining. Pregnancy was confirmed by ultrasound examination of ewes 6 weeks after joining.

Surgery was undertaken to administer the murine monoclonal antibody to foetal lambs and to perform splenectomy. In each case, the ewe was fasted overnight and anaesthesia was induced with thiopentone, and after tracheal intubation, maintained with halothane (1–2%). After laparotomy and exposure of the uterus, the myometrium was incised using cautery allowing the foetal membranes to protrude. Injection of antibody into the foetal peritoneal cavity was performed under direct vision through the foetal membranes. The needle was introduced into the amniotic cavity through the intact myometrium to prevent leakage of fluid. The antibody used was the mouse monoclonal antibody MCM 9 (Beh, 1988), purified by Protein G chromatography from cell culture supernatant and sterilized by filtration (0.22 μm). Another IgG mouse antibody directed against an epithelial component of the follicle-associated epithelium (Du2-69; Landsverk T. and Hein, W.R., personal communication) was also injected intraperitoneally and tissues collected used as a control. Splenectomy was performed as previously described (Press *et al.*, 2001).

Necropsy was performed at the required gestational age following induction of general anaesthesia and the ewe and foetus were euthanased with individual overdose injections of barbiturate. All animal procedures were approved by the Australian National University Animal Experimentation Ethics Committee in accord with the Code of Practice for the Care and Use of Animals for Scientific Purposes of the Australian National Health and Medical Research Council. The collection of frozen tissues from B-cell depleted foetal lambs and splenectomised foetal lambs has been previously described (Press *et al.*, 1996; 2001). Blood was collected from foetal lambs into plain blood collection tubes and allowed to stand at 4°C overnight. Serum was collected and stored at −70°C until analysis.

### Enzyme and Immunohistochemistry

The detection of reactivity for the enzyme 5′nucleotidase has been described previously (Halleraker *et al.*, 1990).

An avidin-biotin-peroxidase immunohistochemical technique was used to stain frozen tissue sections, using a protocol that has been described previously (Gunnes *et al.*, 1999). The primary antibodies used in this procedure were a rabbit polyclonal antibody directed against sheep IgM (μ-chain specific; Cappel Research Products, Durham, NC, USA) and a mouse monoclonal antibody directed against sheep complement receptor-2 (CD21, clone Du2-87-6 (Hein *et al.*, 1998)).

### Estimation of Murine Immunoglobulin in Foetal Sheep Serum

A radial immunodiffusion assay as described previously (Mancini *et al.*, 1964) and a rabbit polyclonal antibody (Dako, ID1797, Glostrup, Denmark) directed against mouse IgG were used. The diameters of the precipitation zones were measured by using a "measuring viewer" (Behringwerke) and micrograms mouse IgG in foetal sheep serum were calculated from a standard curve derived from five dilutions of purified mouse IgG (Dako, ID1915) on each plate. Negative samples contained less than 4 μg mouse IgG per ml (< 0.0043 mg/ml).

### RESULTS

### Definition of Dose of Anti-IgM Antibody Sufficient to Curtail B-cell Development if administered to 63 Day-old Foetal Lambs

As earlier experiments had been confined to the use of a single dose of anti-IgM antibody at a single age, it was not possible to decide whether this dose was close to that which is essential to produce the observed effect or considerably in excess of it. As it was intended in the present series of experiments to compare the effect of a common dose of antibody administered to different foetal lambs at a range of gestational ages, a dose-response estimation was undertaken at the age previously examined. The well-defined appearance of cellular depletion in the lymphoid nodules of the ileal Peyer's patch, which are normally populated by B cells, was used as the basis for determining the outcome of a series of decreasing doses of anti-IgM monoclonal antibody (Table I). In the non-operated control animals, the ileal Peyer's patch between 135–142 dg contains large lymphoid nodules filled with IgM+ B cells (Fig. 1), as did the ileal Peyer's patch of control animals that received an irrelevant mouse IgG antibody at 56 dg (not shown). The nodules also show extensive immunoreactivity for CD21, which is present on B cells and follicular dendritic cells, and enzyme reactivity for 5′nucleotidase, which is present in the extensive stromal cell network in the nodules. In Fig. 1, the influence of anti-IgM antibody administered intraperitoneally at 63 dg on B-cell development in the ileal Peyer's patch is illustrated. Doses of 1.5 and 1.0 mg resulted in depletion of IgM+ B cells from nodules in the ileal Peyer's patch, while doses of 0.5 and 0.25 mg did not completely impede colonisation and expansion of IgM+ B cells in Peyer's patch nodules. On the basis of these observations, a dose of 1 mg was given in all subsequent procedures, unless otherwise indicated.

### The Effects of a Single Dose of 1 mg of Anti-IgM Monoclonal Antibody on Foetal Lambs at a Range of Gestational Ages

It would be anticipated that if the cell population that was being inactivated by the administration of a single inoculum of anti-IgM antibody at 63 dg remained susceptible and accessible thereafter, administration of this antibody at subsequent stages of gestation should exert a similar effect on B-cell development, provided that the concentration of antibody achieved in the compartments containing these cells was adequate. If the size of the cell population to which the monoclonal antibody was targeted exceeded the capacity of that antibody to inactivate them, a gradual decrease in the efficiency with which B-cell development was curtailed might have been anticipated. On the other hand, if for other reasons such as microanatomical inaccessibility or cell surface insusceptibility to binding of the antibody, the target cells were to become resistant to antibody-mediated inactivation, a precipitate decline in the efficacy of inactivation might be observed. In Fig. 2, the effect on ileal Peyer's patch development of a single dose of 1 mg anti-IgM antibody at ages ranging from 70–105 dg is presented.

The intraperitoneal administration of 1 mg of anti-IgM monoclonal antibody up until 84 dg achieved a marked depletion of B cells (Table II). The nodules in the ileal Peyer's patch of foetuses treated at 91 dg, did not show signs of depletion, while the three foetuses treated at 98 dg did show signs of depletion (Fig. 2). At 105 dg, the treated foetuses did not show signs of depletion.

### Body Weight-adjusted Doses of Anti-IgM Monoclonal Antibody extend the Gestational Age at which B-cell Development could be Disrupted

The lack of effect of anti-IgM antibody on the development of B cells in the ileal Peyer's patch, which was present when exposure was delayed until 105 dg, could reflect a decrease of antibody concentration in the foetal extracellular compartment. To assess this possibility, an antibody dose was administered at 98 and 105 dg that had been appropriately adjusted to match the weight of the foetal lamb for a standard 1 mg dose at 63 dg. A marked B-cell depletion of nodules in the ileal Peyer's patch after treatment with the weight-adjusted doses (12.5 mg at 98 dg and 15 mg at 105 dg) was present

TABLE I Dose-response estimation of anti-IgM antibody (αIgM) on B-cell development at 63 days of gestation (dg)

| Treatment | Number of foetuses | Age at operation (dg) | Age at necropsy (dg) | Effect on nodules |
|---|---|---|---|---|
| 1.5 mg αIgM | 1 | 63 | 139 | Depleted |
| 1.0 mg αIgM | 2 | 63 | 139 | Depleted |
| 0.5 mg αIgM | 1 | 63 | 140 | Irregular sizes |
| 0.25 mg αIgM | 2 | 63 | 139 | Irregular sizes |
| 1.0 mg mouse IgG | 4 | 56 | 140 | Normal |
| Control | 8 | – | 135–142 | Normal |

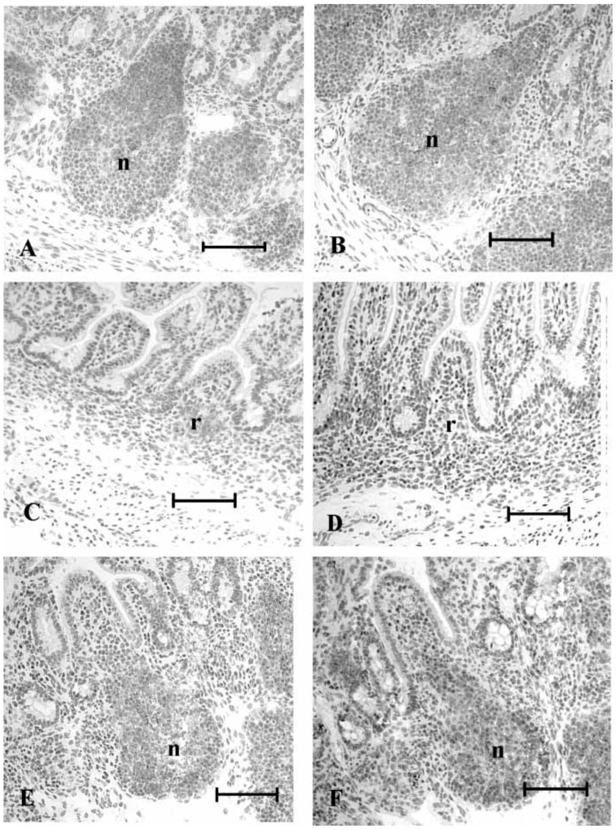

FIGURE 1 B-cell development in the pre-natal ileal Peyer's patch of lambs following intraperitoneal administration of various doses of anti-IgM antibody at 63 days of gestation (dg). Normal untreated control lambs possess large nodules (n) that show staining (brown) for (A) CD21 and (B) ovine IgM. Following administration of 1.5 mg anti-IgM antibody, small rudimentary nodules (r) are present that show staining for (C) CD21 but not for (D) ovine IgM. Following administration of 0.5 mg anti-IgM antibody, large nodules (n) are present that show staining for (E) CD21 and (F) ovine IgM. Bar = 50 μm. Frozen sections.

at 137 and 142 d g, respectively (Fig. 3, Table III). Thus, an increase in the dose of anti-IgM antibody to restore the dose/body weight ratio to that applying when 1 mg was administered to 63 dg foetal lambs impeded B-cell development in the ileal Peyer's patch.

### Failure of Splenectomy at 56 dg to restore the Capacity of 1 mg Anti-IgM Antibody administered at 105 dg to Deplete the Ileal Peyer's Patch of B Cells

If the inability of anti-IgM antibody to perturb B-cell development reflects an inadequacy of antibody concentration in the appropriate tissue compartments, then

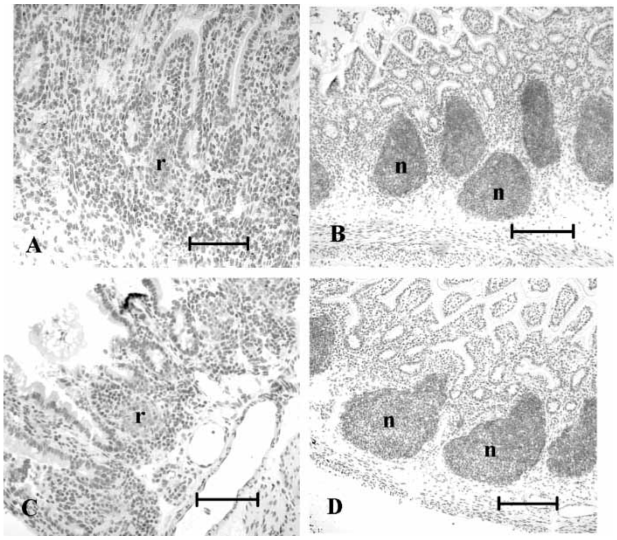

FIGURE 2 B-cell development in the pre-natal ileal Peyer's patch of lambs following intraperitoneal administration of 1 mg anti-IgM antibody at a range of gestational ages (dg). Following administration of anti-IgM antibody, immunohistochemical staining for CD21 (brown) shows that: (A) At 70 dg, small rudimentary nodules (r) are present. Bar = 50 μm. (B) At 91 dg, large nodules (n) are present. Bar = 100 μm. (C) At 98 dg, small rudimentary nodules (r) are present. Bar = 50 μm. (D) At 105 dg, large nodules (n) are present. Bar = 100 μm. Frozen sections.

reducing the size of the B-cell compartment in foetal lambs may restore the capacity of lower doses of anti-IgM to impede B-cell development at later stages of gestation. Alternatively, otherwise susceptible populations may be sequestered in the spleen. To investigate these possibilities, splenectomies were performed in combination with the intraperitoneal administration of anti-IgM antibody. Previous studies have shown that the spleen is a major site of early B-cell accumulation (Press *et al.*, 1993) and while splenectomy at 56 dg does not prevent B-cell development in the ileal Peyer's patch (Press *et al.*, 2001), its removal would be expected to eliminate an important micro-environment for the expansion of the B-cell compartment in foetal lambs.

Foetal lambs were splenectomised at 56 dg and then inoculated with 1 mg anti-IgM antibody at 56 dg or at 105–106 dg (Table IV). At necropsy, the ileal Peyer's patch did not show a marked depletion of B cells when foetal lambs were inoculated with anti-IgM antibody at 105–106 dg in splenectomised animals, although two individuals showed signs of irregular nodular development (Fig. 4).

TABLE II Effect of anti-IgM antibody (αIgM) administered at 7-day intervals from 70 to 105 days of gestation on B-cell development

| Treatment | Number of foetuses | Age at operation (days of gestation) | Age at necropsy (days of gestation) | Effect on nodules |
|---|---|---|---|---|
| 1.0 mg αIgM | 1 | 70 | 140 | Depleted |
| 1.0 mg αIgM | 1 | 77 | 135 | Depleted |
| 1.0 mg αIgM | 2 | 84 | 139 | Depleted |
| 1.0 mg αIgM | 2 | 91 | 139, 140 | Normal |
| 1.0 mg αIgM | 3 | 98 | 139 | Depleted |
| 1.0 mg αIgM | 2 | 105 | 141 | Normal |

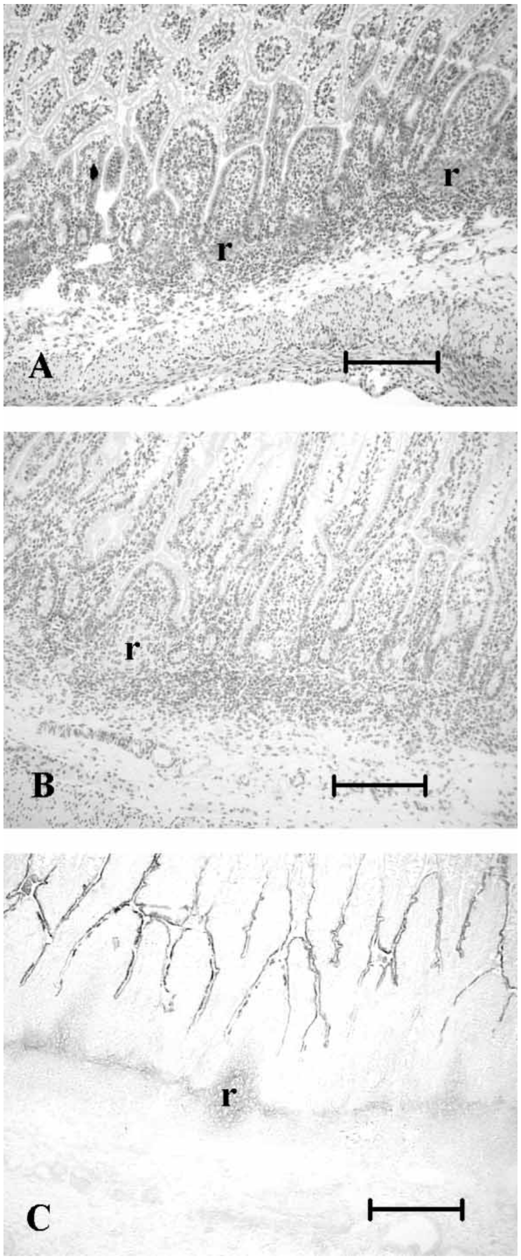

FIGURE 3 B-cell development in the pre-natal ileal Peyer's patch of lambs following body weight-adjusted doses of anti-IgM antibody. (A) Following administration of 15 mg anti-IgM antibody at 105 days of gestation (dg), small rudimentary nodules (r) are present showing staining for CD21 (brown). (B) Following administration of 12.5 mg anti-IgM antibody at 98 dg, small rudimentary nodules (r) are present that do not show staining for ovine IgM. (C) Following administration of 12.5 mg anti-IgM antibody at 98 dg, small rudimentary nodules (r) are present showing enzyme reactivity for 5′nucleotidase. Bar = 100 μm. Frozen sections.

**Persistence of Mouse Immunoglobulin in the Blood of Foetal Lambs following Intraperitoneal Administration**

To determine the persistence of mouse immunoglobulin in the blood of foetal lambs following intraperitoneal administration, anti-IgM antibody was administered at around 82–85 dg and serum was collected at necropsy at times ranging from 1 to 21 days after administration (age range at necropsy 84–105 dg). Serum was also collected at necropsy of other experimental and control foetal lambs in the present series of experiments including additional foetuses that received high doses of anti-IgM antibody. Three additional foetuses were given antibody at 98 dg (12.5 mg) and three at 103 dg (15 mg)(Table V).

Following administration of 1 mg of anti-IgM antibody, the average estimated concentration of mouse immunoglobulin in serum was highest 3 days after administration and was still detectable in serum 14 days after administration. Murine immunoglobulin was not detectable at 21 days or at longer time intervals after administration. With the administration of larger amounts of antibody, high concentrations of murine immunoglobulin were detected for longer periods after administration, up to 45 days after administration (Table V).

### DISCUSSION

This study examined the impact of an anti-IgM monoclonal antibody, administered to foetal lambs at a range of gestational ages and in fixed and body-weight adjusted doses, on the development of B cells in the ileal Peyer's patch. The intraperitoneal injection of 1 mg antibody at 63 dg has been shown to result in a marked depletion of B cells in the ileal Peyer's patch, which persisted until the time of necropsy just prior to birth (Press *et al.*, 1996). The present study showed that this dose of antibody was effective in producing B-cell depletion if administered at gestational ages ranging from 56 to 84 dg. However, beyond 91 dg, the effect appeared to be inconsistent and was absent at 105 dg. The antibody-mediated depletion of B cells from the ileal Peyer's patch was restored when the dose of antibody was increased sufficiently to ensure that parity with foetal body weight was maintained. Thus, the present study shows that the basis of anti-IgM interception of B-cell development in the ileal Peyer's patch of foetal lambs is the delivery of an effective concentration of antibody, which is presumably adequate to saturate receptors on a significant proportion

TABLE III Effect of weight-adjusted dose of anti-IgM antibody (αIgM) on B-cell development

| Treatment | Number of foetuses | Age at operation (days of gestation) | Age at necropsy (days of gestation) | Effect on nodules |
|---|---|---|---|---|
| 12.5 mg αIgM | 1 | 98 | 137 | Depleted |
| 15.0 mg αIgM | 1 | 105 | 142 | Depleted |

TABLE IV Effect of splenectomy (Splx) and 1.0 mg anti-IgM antibody (αIgM) administration on B-cell development. Age of foetal lambs is given as days of gestation

| Treatment | Number of foetuses | Age at splenectomy | Age at αIgM admin. | Age at necropsy | Effect on nodules |
|---|---|---|---|---|---|
| αIgM only | 1 | – | 56 | 142 | Depleted |
| Splx + αIgM | 1 | 56 | 56 | 142 | Depleted |
| Splx + αIgM | 2 | 56 | 105 | 138–140 | Irregular sizes |
| Splx + αIgM | 2 | 56 | 105–106 | 138–140 | Normal |
| αIgM only | 1 | – | 105 | 141 | Normal |

of the target lymphocyte population. This study further shows that the expanding Peyer's patch nodules present at 105 dg do not shield the proliferating B cells from the effect of a sufficient concentration of anti-IgM antibody.

The present study did not pursue the effect of anti-IgM antibody on the developed ileal Peyer's patch but rather focused on B cells that are present in the foetus up to 105 dg. The ileal Peyer's patch is responsible for the production of the vast majority of B cells in sheep and achieves an anatomical distribution and appearance of the postnatal organ from about 115 dg (Press *et al.*, 1992). Active lymphopoiesis commences in the ileal Peyer's patch at around 100 dg (Reynolds and Morris, 1983) but prior to this time IgM+ B cells are present in a number of sites including the spleen (Maddox *et al.*, 1987a), thymus (Maddox *et al.*, 1987b), lymph nodes (Maddox *et al.*, 1987c; Press *et al.*, 1993), circulation (Symons and Binns, 1975) and other gut-associated lymphoid tissues (Aleksandersen *et al.*, 1991). It should be noted that in sheep lymphopoiesis in the bone marrow is minor and the liver is not a source of IgM+ cells (Miyasaka and Morris, 1988).

The spleen has been proposed as a source of B cells that colonise the ileal Peyer's patch in cattle (Lucier *et al.*, 1998) but a recent study on the effect of early foetal splenectomy in sheep found that the spleen was not essential for the colonisation and development of

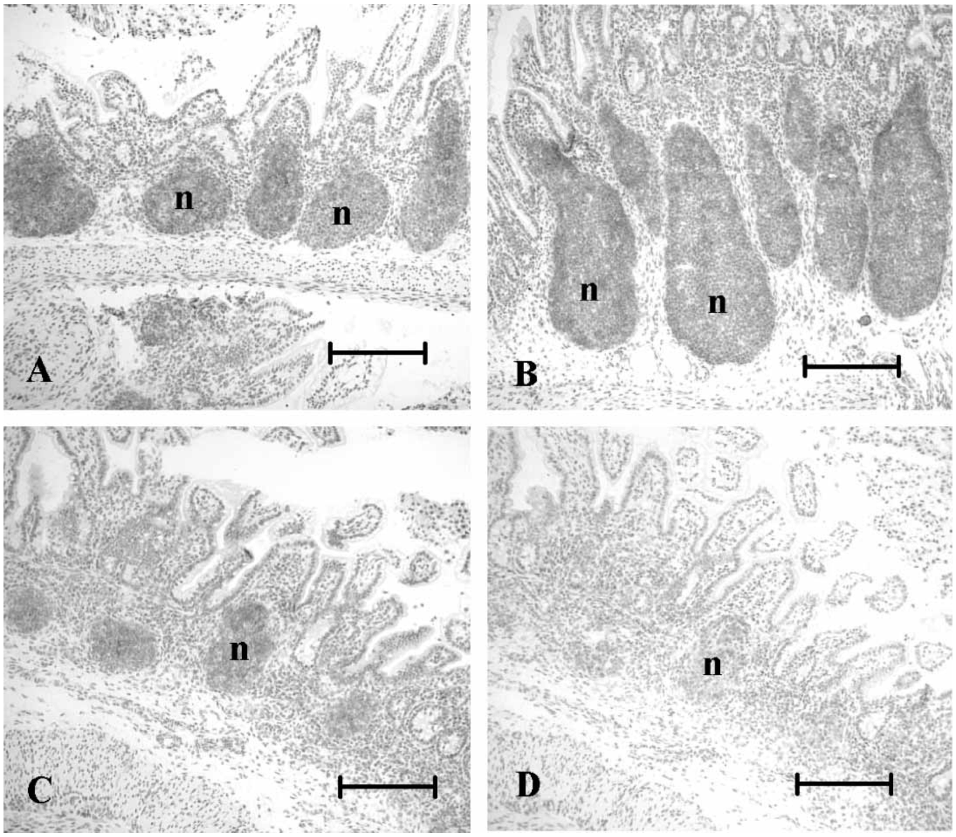

FIGURE 4 B-cell development in the pre-natal ileal Peyer's patch of lambs following splenectomy at 56 days of gestation (dg) and intraperitoneal administration of 1 mg anti-IgM antibody at 105 dg. Large nodules (n) showing staining for CD21 (brown) are present (A) following administration of anti-IgM antibody at 105 dg only and (B) following splenectomy at 56 dg and administration of anti-IgM antibody at 105 dg. In another foetus that was splenectomised at 56 dg and received anti-IgM antibody at 105 dg, dispersed nodules (n) of irregular size were present that showed staining for (C) CD21, and (D) ovine IgM (brown). Bar = 100 μm. Frozen sections.

TABLE V Estimated concentration (μg/ml) of mouse immunoglobulin in serum from foetal lambs at various times after intraperitoneal administration of a single bolus of antibody (mg)

| Number of days after administration | Number of foetuses | Amount of antibody administered (mg) | Average estimated concentration (μg/ml) |
|---|---|---|---|
| 1 | 2 | 1 | 3.4 |
| 3 | 2 | 1 | 5.8 |
| 4 | 2 | 1 | 5.5 |
| 7 | 2 | 1 | 4.3 |
| 14 | 2 | 1 | 4.1 |
| 21 | 2 | 1 | 0 |
| 35 | 1 | 1 | 0 |
| 49 | 2 | 1 | 0 |
| 55,58 | 2 | 1 | 0 |
| 70 | 1 | 1 | 0 |
| 86 | 3 | 1 | 0 |
| 40 | 3 | 12.5 | 7.8 |
| 45 | 1 | 12.5 | 8.0 |
| 37 | 1 | 15 | 15.8 |
| 40 | 2 | 15 | 13.1 |
| 44 | 1 | 15 | 8.0 |
| 84 (Mouse IgG) | 3 | 1 | 0 |
| Control 104 dg | 1 | 0 | 0 |
| Control 125 dg | 1 | 0 | 0 |
| Control 140 dg | 2 | 0 | 0 |

the ileal Peyer's patch (Press *et al.*, 2001). However, prior to the colonisation of the ileal Peyer's patch, the spleen is a significant site of B-cell accumulation (Press *et al.*, 1993) and findings in splenectomised foetuses suggest that splenic B-cell populations influence ileal Peyer's patch development (Press *et al.*, 2001). The present study found that splenectomy at 56 dg followed by administration of 1 mg anti-IgM antibody at 105 dg did not prevent B-cell development in the ileal Peyer's patch, although some foetuses showed signs that development was affected (Fig. 4). These experiments indicate that in the absence of the spleen, other tissue microenvironments are able to support and promote the expansion of B-cell populations above the arbitrary threshold imposed by anti-IgM administration that is necessary for the subsequent development of the ileal Peyer's patch. The localisation and distribution of B cells in splenectomised foetal sheep prior to establishment of the ileal Peyer's patch has not been investigated but further characterisation of lymphocyte populations in these animals could shed light on the relative capacity of other lymphoid tissue microenvironments to support B-cell development and their influence on immunoglobulin V-gene usage and diversification in foetal sheep (Hein and Dudler, 1998; 1999).

An important finding in the present study was the estimation of the concentration of mouse immunoglobulin persisting in foetal sheep serum at various times after intraperitoneal administration. This series of experiments showed that high concentrations of anti-IgM antibody are in the serum for up to two weeks after administration of a dose of 1 mg and that high concentrations are maintained for longer periods of time if foetal weight-adjusted doses were administered. These findings are consistent with the low catabolic rate of ovine gamma globulin demonstrated in foetal ruminants and suggest that lysozymal enzymes present in the liver, spleen, lymph nodes and plasma have a limited capacity for protein degradation (Fukumoto and Brandon, 1985).

It is interesting to note that no murine immunoglobulin was detectable in serum 21 days or longer after the administration of 1 mg of antibody. If, as other findings in this study suggest, the effect of anti-IgM administration is related to the concentration of mouse immunoglobulin in serum, then the effect of administration of antibody at 56 dg can at most have been present until 77 dg. The investigation of B-cell depleted foetal sheep has shown that rudimentary follicles containing follicular dendritic cells are present in the ileum just prior to birth (Press *et al.*, 1998) and that B-cell populations, albeit reduced, are present in the spleen, lymph nodes and jejunal Peyer's patch (Press *et al.*, 1996). The question is then why in the absence of anti-IgM antibody, do the B-cell populations that are present in B-cell depleted foetal lambs fail to colonise the emerging lymphoid nodules of the ileal Peyer's patch in the remaining 73 days of gestation?

One possibility is that sufficient new B cells are not produced and/or those remaining are incapable of colonisation. Reynaud *et al.* (1991) observed a limited pattern of light chain rearrangement in the ileal Peyer's patch of sheep, which was interpreted to indicate that each ileal Peyer's patch nodule had been colonised by a small number of precursor cells having rearranged one light chain allele. Similar findings in the bursa of Fabricius of the chicken have been used to argue that rearrangement only occurs during a narrow window of development (Reynaud *et al.*, 1992). Thus, if on-going differentiation of B cells from stem cells does not occur in sheep and anti-IgM administration induces apoptosis or switches off proliferation in existing B cells (Griebel *et al.*, 1991; Motyka *et al.*, 1995), then sufficient numbers of appropriate colonising populations may not be present. An investigation of immunoglobulin gene rearrangement and proliferation and apoptosis in normal and B-cell depleted foetal sheep would provide information on whether B-cell populations emerge after anti-IgM administration and whether B cells gain entry to the developing nodules in anti-IgM treated foetal sheep. The administration of anti-IgM antibody at gestational ages earlier than 56 days should also be undertaken.

Another possible explanation for the observation of Reynaud *et al.* (1991) that is also consistent with the findings of the present study is that the ileal Peyer's patch can only be colonised during a limited period of gestation. An extensive series of experiments with chick-quail chimeras has shown that the bursa of Fabricius can only be colonised at specific times during gestation (le Douarin *et al.*, 1984). Thus, anti-IgM antibody administration may function to suppress, impede or eliminate B-cell populations during the crucial period in which the ileal Peyer's patch is capable of being colonised. New B-cell populations may be produced or surviving populations may expand after anti-IgM administration but these B cells are unable to gain entry to the privileged microenvironment of the ileal Peyer's patch. The presence of nodules of irregular sizes following administration of a sub-optimal dose (0.5 and 0.25 mg) of anti-IgM antibody or in some individuals that had been splenectomised and subsequently given anti-IgM antibody may reflect a disturbance of the colonisation of nodules.

The present series of experiments shows that the basis of anti-IgM interception of B-cell development in the ileal Peyer's patch is the availability of adequate concentrations of intraperitoneally administered antibody. However, the study also shows that foetal sheep were able to clear a 1 mg bolus of anti-IgM antibody within 21 days of administration. The inability of B-cell populations to colonise the ileal Peyer's patch during the extended period remaining after the clearance of a single bolus of antibody indicates that further studies are needed to investigate the nature of B cells that colonise Peyer's patch nodules in foetal sheep.

### Acknowledgements

The authors acknowledge the skilful technical assistance of Karen King, Bernie Barancewicz, Grethe M. Johansen, Laila Aune, Inger Rudshaug and Tor Mellem. This study was supported in part by grants from the Norwegian Research Council.

### References

Aleksandersen, M., Nicander, L. and Landsverk, T. (1991) "Ontogeny, distribution and structure of aggregated lymphoid follicles in the large intestine of sheep", *Developmental and Comparative Immunology* **15**, 413–422.

Alt, F.W., Blackwell, T.K. and Yancopoulos, G.D. (1987) "Development of the primary antibody repertoire", *Science* **238**, 1079–1087.

Beh, K.J. (1988) "Monoclonal antibodies against sheep immunoglobulin light chain, IgM and IgA", *Veterinary Immunology and Immunopathology* **18**, 19–27.

Cooper, M.D., Kearney, J.F., Gathings, W.E. and Lawton, A.R. (1980) "Effects of anti-immunoglobulin antibodies on the development and differentiation of B cells", *Immunological Reviews* **52**, 29–53.

Fukumoto, T. and Brandon, M.R. (1985) "Immunoglobulin metabolism in the sheep fetus", In: Morris, B. and Miyasaka, M., eds, Immunology of the Sheep (Editiones Roche, Basle, Switzerland), pp 127–154.

Gordon, J. (1979) "The B lymphocyte-deprived mouse as a tool in immunobiology", *Journal of Immunological Methods* **25**, 227–238.

Griebel, P.J., Davis, W.C. and Reynolds, J.D. (1991) "Negative signaling by surface IgM on B cells isolated from ileal Peyer's patch follicles of sheep", *European Journal of Immunology* **21**, 2281–2284.

Gunnes, G., Jörundsson, E., Press, C.McL., Skjerve, E., Ulvund, M. and Landsverk, T. (1999) "Increased γδ T-cell populations in draining lymph nodes of lambs during the elicitation phase of dinitrochlorobenzene-induced contact hypersensitivity", *Developmental and Comparative Immunology* **23**, 665–675.

Halleraker, M., Landsverk, T. and Nicander, L. (1990) "Organization of ruminant Peyer's patches as seen with enzyme histochemical markers of stromal and accessory cells", *Veterinary Immunology and Immunopathology* **26**, 93–104.

Hein, W.R. and Dudler, L. (1998) "Diversity of Ig light chain variable region gene expression in fetal lambs", *International Immunology* **10**, 1251–1259.

Hein, W.R. and Dudler, L. (1999) "Diversification of sheep immunoglobulins", *Veterinary Immunology and Immunopathology* **72**, 17–20.

Hein, W.R., Dudler, L., Marston, W.L., Landsverk, T., Young, A.J. and Avila, D. (1998) "Ubiquitination and dimerization of complement receptor type 2 on sheep B cells", *Journal of Immunology* **161**, 458–466.

le Douarin, N.M., Dieterlen-Lievre, F. and Oliver, P.D. (1984) "Ontogeny of primary lymphoid organs and lymphoid stem cells", *American Journal of Anatomy* **170**, 261–299.

Lucier, M.R., Thompson, R.E., Waire, J., Lin, A.W., Osborne, B.A. and Goldsby, R.A. (1998) "Multiple sites of Vλ diversification in cattle", *Journal of Immunology* **161**, 5438–5444.

Maddox, J.F., Mackay, C.R. and Brandon, M.R. (1987a) "Ontogeny of ovine lymphocytes. II. An immunohistochemical study on the development of T lymphocytes in the sheep fetal spleen", *Immunology* **62**, 107–112.

Maddox, J.F., Mackay, C.R. and Brandon, M.R. (1987b) "Ontogeny of ovine lymphocytes. I. An immunohistochemical study on the development of T lymphocytes in the sheep embryo and fetal thymus", *Immunology* **62**, 97–105.

Maddox, J.F., Mackay, C.R. and Brandon, M.R. (1987c) "Ontogeny of ovine lymphocytes. III. An immunohistochemical study on the development of T lymphocytes in the sheep fetal lymph nodes", *Immunology* **62**, 113–118.

Mancini, G., Vaerman, J.-P., Carbonara, A.O. and Heremans, J.F. (1964) "A single-radial-diffusion method for the immunological quantitation of proteins", In: Peeters, H., ed, Proteins of the Biological Fluids (Elsevier, Amsterdam), pp. 370–373.

Miyasaka, M. and Morris, B. (1988) "The ontogeny of the lymphoid system and immune responsiveness in sheep", *Progress in Veterinary Microbiology and Immunology* **4**, 21–55.

Motyka, B., Bhogal, H.S. and Reynolds, J.D. (1995) "Apoptosis of ileal Peyer's patch B cells is increased by glucocorticoids or anti-immunoglobulin antibodies", *European Journal of Immunology* **25**, 1865–1871.

Opstelten, D. and Osmond, D.G. (1985) "Regulation of pre-B cell proliferation in bone marrow: immunofluorescence stathmokinetic studies of cytoplasmic μ chain-bearing cells in anti-IgM-treated mice, hematologically deficient mutant mice and mice give sheep red blood cells", *European Journal of Immunology* **15**, 599–605.

Press, C.McL., Halleraker, M. and Landsverk, T. (1992) "Ontogeny of leukocyte populations in the ileal Peyer's patch of sheep", *Developmental and Comparative Immunology* **16**, 229–241.

Press, C.McL., Hein, W.R. and Landsverk, T. (1993) "Ontogeny of leucocyte populations in the spleen of fetal lambs with emphasis on the early prominence of B cells", *Immunology* **80**, 598–604.

Press, C.McL., Reynolds, J.D., McClure, S.J., Simpson-Morgan, M.W. and Landsverk, T. (1996) "Fetal lambs are depleted of $IgM^+$ cells following a single injection of an anti-IgM antibody early in gestation", *Immunology* **88**, 28–34.

Press, C.McL., Reynolds, J.D., McClure, S.J. and Landsverk, T. (1998) "Development of accessory cells in B-cell compartments is retarded in B-cell-depleted fetal sheep", *Developmental Immunology* **6**, 223–231.

Press, C.McL., McCullagh, P. and Landsverk, T. (2001) "Effect of early foetal splenectomy on prenatal B-cell development in sheep", *Immunology* **102**, 131–136.

Reynaud, C.-A., Mackay, C.R., Müller, R.G. and Weill, J.-C. (1991) "Somatic generation of diversity in a mammalian primary lymphoid organ: The sheep ileal Peyer's patches", *Cell* **64**, 995–1005.

Reynaud, C.-A., Imhof, B.A., Anquez, V. and Weill, J.-C. (1992) "Emergence of committed B lymphoid progenitors in the developing chicken embryo", *EMBO Journal* **11**, 4349–4358.

Reynolds, J.D. and Morris, B. (1983) "The evolution and involution of Peyer's patches in fetal and postnatal sheep", *European Journal of Immunology* **13**, 627–635.

Symons, D.B. and Binns, R.M. (1975) "Immunoglobulin-bearing lymphocytes; their demonstration in adult sheep and ontogeny in the sheep foetus", *International Archives of Allergy and Applied Immunology* **49**, 658–669.

